# Methods in Electricity

**Published:** 1887-06

**Authors:** F. T. Paine

**Affiliations:** Comanche, Texas


### METHODS IN ELECTRICITY.

*By F. T. Paine, M. D., Comanche, Texas.*

For Daniel's Texas Medical Journal.

IN compliance with promises to various members of "Texas State Medical Association," I will say that the instrument with which I have performed all my various experiments for the last three years is Dr. Jerome Kidder's No. 5 Tip Battery, than which I desire no better, unless it may be his No. 1, which is large, with some improvements and conveniences.

One change I would make with any instrument now in the market, that is, substitute conducting cords of unequal lengths. One cord should be 2 yards, the other 3 yards long. When treating inflammations and fevers, I place the short cord on the positive post; the long cord on the negative post of the battery ; thus placing my patient entirely under the influence of positive electricity, and by these means reduced the temperature. The reverse position of the cords produces a stimulating effect, and raises the temperature, the philosophy of which I may give later.

Again the electrodes supplied by the manufacturers are too small for general use. I have several made by the tin or coppersmith. A copper plate, 4 x 8, or 12 inches for the feet, another 2 x 4, or 6 inches for back, are indispensible. The back over the spinal column, is the most insensible part of the body to the Faradic current, and being directly over and near to the origin of all the spinal nerves, is therefore the best location for the indifferent and stabile electrode, the other, or active, or labile electrode can of course be used whenever necessary. The whole length of spine from the atlas to os coxygis is normally insensible to the current, and if you find it otherwise, it is an evidence of abnormality, and electric treatment will restore the normal insensibility, when the spinal cord shall have been restored to a normal state ; for the disease is internal, not external,—this state obtains in some of the paralyses.

Of course, these electrodes must be well covered with soft, thick cloth, canton flannel or a stocking leg answers well.

An other electrode I find valuable is made by placing a small piece of covered metal under a wristlet on the arm, and attaching a cord to the metal, converts the hand into a valuable instrument for diagnosis and in many cases for the treatment of the head and face; the genitalia of females, and always for children. But a word of caution will be well here. I have a personal experience of the loss of 20 lbs. of extra obesity in 6 or 8 months incidental use of this electrode. "A word to the wise". Of course the wire brush, vaginal and rectal electrodes, will be necessary. I use the last two named not only for the purposes for which they were designed but much more in the treatment of neuralgia, rheumatism and other painful diseases. Before closing this paper, I cannot refrain from a few remarks as to the operator himself.

It requires faith, patience and perseverance to commence with, and then more hard study than most of the profession have devoted to any one or perhaps all other branches. If you suppose that, because the machine is automatic, all you have to do is to wind it up, give it a flip and let her rip, you are mistaken, your money and time misapplied and you a failure from the beginning and will only bring reproach upon yourself and this branch of your profession. The books on the subject are numerous, but the so-called science of electro therapeutics in "confusion worse confounded." — The work best suited to one who has but little time to devote to this branch, is CLARK'S work, by A. W. Tipton, where you will find the science in a nut shell, with prescriptions cut and dried; this is also best for a lazy man.

One more idea, a little late, but none the less important—never let patients handle the electrodes or hold them in their hands. The hands are always sensitive and no criterion for any other part, and nervous persons become alarmed, so that it is difficult afterwards to control them.

Use mild currents, and never let the treatment be painful, unless absolutely necessary, as it will be in rheumatism and neuralgia, of which more anon,—the editor consenting.

---

The North Texas Medical Association held its regular semi-annual meeting at Bonham, Texas, on the 14th, inst., (June, '87), a three days meeting. Dr. J. M. Fort, of Paris, President, presiding. Dr. G. R. Clayton, Secretary. We had no representative present, and regret not to be able to give a report of the meeting.

